# Analysis of clinical and genetic characteristics in 10 Chinese individuals with Cornelia de Lange syndrome and literature review

**DOI:** 10.1002/mgg3.1471

**Published:** 2020-08-27

**Authors:** Chen Liu, Xiaoying Li, Jing Cui, Rui Dong, Yvqiang Lv, Dong Wang, Haiyan Zhang, Xiaomei Li, Zilong Li, Jian Ma, Yi Liu, Zhongtao Gai

**Affiliations:** ^1^ Department of Neonatology Pediatric Research Institute Qilu Children's Hospital Cheeloo College of Medicine Shandong University Jinan China; ^2^ Department of Neonatology Pediatric Research Institute Jinan Children's Hospital Jinan China; ^3^ Department of Neonatology Qilu Children's Hospital of Shandong University Jinan China; ^4^ Pediatric Research Institute Qilu Children's Hospital of Shandong University Jinan China

**Keywords:** Chinese CdLS individuals, Cornelia de Lange syndrome, genotype‐phenotype relationship, variant

## Abstract

**Background:**

Cornelia de Lange syndrome (CdLS) is a rare congenital developmental disorder with variable multisystem involvement and genetic heterogeneity. We aimed to analyze the clinical and genetic characteristics of Chinese individuals with CdLS.

**Methods:**

We collected data regarding the neonatal period, maternal status, clinical manifestation, including facial dimorphisms and development, and follow‐up treatment for individuals diagnosed with CdLS. In individuals with suspected CdLS, high‐throughput sequencing, Sanger sequencing, and real‐time qualitative PCR were used to verify the diagnosis.

**Results:**

Variants, including six that were novel, were concentrated in the *NIPBL* (70%), *HDAC8* (20%), and *SMC3* (10%) genes. We found two nonsense, three splicing, and two deletion variants in *NIPBL*; a missense variant and an absence variant in *HDAC8*; and a missense variant in *SMC3*. Eleven cardinal features of CdLS were present in more than 80% of Chinese individuals. Compared with non‐Chinese individuals of diverse ancestry, there were significant differences in the clinical characteristics of eight of these features.

**Conclusion:**

Six novel pathological variants were identified; thus, the study expanded the gene variant spectrum. Furthermore, most cardinal features of CdLS found in Chinese individuals were also found in individuals from other countries. However, there were significant differences in eight clinical features.

## Graphical abstract

1. My article is the most number reports in China on accurate diagnosis of CdLS in the level of gene, including 6 novel gene mutations of new case reports.
2. The successful case of growth hormone application was added to the exisiting literature, providing a new approach for the treatment of CdLS.
3. The impact is expanding the gene spectrum of CdLS and the relationship between genotype and phenotype for CdLS.

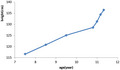

## 1 INTRODUCTION

Cornelia de Lange syndrome (CdLS; Online Mendelian Inheritance in Man (OMIM) 122470, 300590, 300882, 610759, and 614701), is an established multiple malformation syndrome typically involving physical, cognitive, and behavioral characteristics (Boyle, Jespersgaard, Brøndum‐Nielsen, Bisgaard, & Tümer, 2014; Kline et al., 2018). It is autosomal dominant or X‐linked dominant disorder with an estimated incidence of 1 in 10,000–30,000 live births (Mannini, Cucco, Quarantotti, Krantz, & Musio, 2013). The syndrome is named after the Dutch pediatrician Cornelia de Lange, who recognized the developmental disorder in two infants during 1933 (Goodban, 1993). The clinical characteristics of classical CdLS include distinctive craniofacial features, intrauterine and postnatal growth retardation, limb deformities, and severe intellectual disability with a mean IQ of 53 (Kline et al., 2007).

Whole‐exome sequencing (WES) has revolutionized genomic investigations over the past decade along with the molecular diagnosis of individuals with growth retardation and intellectual disabilities. Molecular investigations have attributed the etiology of CdLS to genetic variants of structural or regulatory components of the cohesin complex. Seven genes associated with CdLS have been identified, namely, nipped‐B‐like protein (*NIPBL*), structural maintenance of chromosomes 1A (*SMC1A*) and 3 (*SMC3*), double‐strand break repair protein rad21 homolog (*RAD21*), bromodomain‐containing protein 4 (*BRD4*), histone deacetylase 8 (*HDCA8*), and ankyrin repeat Domain 11 (*ANKRD11*). The most prevalent is the *NIPBL* gene, which is found in about 70% of individuals (Kline et al., 2018; Watrin, Kaiser, & Wendt, 2016).

The International CdLS Consensus Group, comprising 43 participants from 30 institutions in nine countries, published the first international consensus on CdLS in Nature Review during 2018. The consensus elaborated clinical diagnostic criteria for classical and nonclassical CdLS phenotypes, molecular investigations, long‐term management, and care planning (Huisman et al., 2017; Kline et al., 2007).

Here, we applied WES to 10 children with suspected CdLS, and found genetic mutations of the *NIPBL*, *HDAC8*, and *SMC3* genes in seven, two, and one of the individuals, respectively. We also found six novel variants in the *NIPBL* genes. We analyzed the relationships between genotypes and phenotypes to provide a basis for clinical diagnosis and genetic counseling.

## 2 MATERIALS AND METHODS

### 2.1 Ethical compliance

All patient referrals and genetic tests were voluntary. All study procedures were conducted according to the Declaration of Helsinki (2013 amendment) and the Medical Ethics Committee at Qilu Children's Hospital of Shandong University (reference number: ETYY‐2020217). The parents of the individuals provided written informed consent to clinical and laboratory examinations before starting the study. All information was rendered innominate before submission.

### 2.2 Individuals and samples

We enrolled 10 children with suspected CdLS according to the clinical diagnostic criteria in the first international consensus statement (Kline et al., 2018), from 10 unrelated Chinese families at a neonatal intensive care unit and a child care clinic between September 2015 and August 2019. Genetic variants were detected at the Pediatric Research Institute at Qilu Children's Hospital of Shandong University. Pediatricians collected maternal history, clinical features, imaging and laboratory findings, and clinical management and treatment information about the individuals.

### 2.3 Validation of pathological variants

We sequenced the *NIPBL*, *SMC1A*, *SMC3*, *RAD21*, *BRD4*, and *HDAC8* genes of CdLS in peripheral blood lymphocytes from the probands using target region sequence capture and high‐throughput gene sequencing. The variants nomenclature followed the HGVS nomenclature guidelines (http://www.hgvs.org/mutnomen). The GenBank reference sequences mentioned in this study were *NIPBL* (NG_006987.2), *HDAC8* (NG_015851.1), and *SMC3* (NG_012217.1) using the version of GRCh37/hg19. The results were compared with the reference sequences of genes associated with CdLS, and variants were searched in the 1000 Genome, human ExAC integrated, Clin Var and Human Gene Mutation (HGMD®) databases.

### 2.4 Literature review and statistical analysis

We searched the PubMed, Wanfang, and China National Knowledge Infrastructure online literature databases for articles that reported cases of CdLS in the Chinese population. Only articles that included genetic sequencing diagnosis and in which the diagnostic criteria were consistent with the American College of Medical Genetics and Genomics (ACMG) guidelines were retained for analysis. We compared the phenotypic findings of these studies with those from a study by Dowsett et al. (2019), which collated clinical data from CdLS individuals of African, Asian, Latin American, and Middle Eastern descent. Findings were compared with chi‐squared tests using the software. Differences were considered statistically significant when the *P* value was less than 0.05. All results are reported with 95% confidence interval.

## 3 RESULTS

### 3.1 Neonatal period and maternal status

Table 1 summarizes the characteristics of the neonatal period and maternal status of the 10 individuals (males, *n* = 5; females, *n* = 5) with CdLS. The neonates were delivered at an average gestational age of 37.2 ± 4.3 weeks, the average birth weight was 2235 ± 460 g, and all had intrauterine growth restriction (IUGR). The mean age at diagnosis was 22 months (five neonates were diagnosed during the neonatal period). The ratio of cesarean section to spontaneous vaginal delivery was 7:3. At the time of delivery, three mothers with individuals of CdLS had been exposed to amniotic fluid pollution and five had polyhydramnios. One infant (individual 9) suffered from perinatal asphyxia. The average maternal age at conception was 29 ± 4 years, and eight of the women were healthy during pregnancy. One woman developed an upper respiratory tract infection during the third mouth of pregnancy, and another woman had hypothyroidism. Parental consanguinity or a positive family history were not associated in any of the individuals.

**Table 1:** Neonatal period and maternal condition

	Gender	Low birth weight		Premature infant	Twins	Age at diagnosis			Delivery pattern		Amniotic fluid			Maternal health status	
		＜10th percentile	＜3rd percentile			Newborn	1–3 years	4–10 years	Spontaneous vaginal delivery	Cesarean section	Pollution	Polyhydramnios	Asphyxia	Healthy	Early pregnancy infection
1	Female	−	+	−	+	+	−	−	−	+	+	−	−	+	−
2	Male	+	−	+	−	+	−	−	−	+	+	−	−	_	−
3	Male	−	+	−	−	−	−	+	−	+	−	+	−	_	+
4	Female	−	+	+	−	−	−	+	+	−	−	−	−	+	−
5	Female	+	−	−	−	+	−	−	−	+	−	+	−	+	−
6	Male	+	−	−	−	−	+	−	−	+	−	+	−	+	−
7	Female	−	+	−	−	−	+	−	−	+	+	−	−	+	−
8	Male	−	+	−	−	+	−	−	+	−	−	−	−	−	+
9	Female	−	+	−	−	−	+	−	−	+	−	+	+	+	−
10	Male	+	−	+	−	+	−	−	+	−	−	+	−	+	−

### 3.2 Clinical manifestations

The phenotypes were classified according to the international consensus as main and suggestive features, and all 10 individuals scored ≥11. Table 2 lists the clinical manifestations of the individuals, including prenatal and postnatal growth retardation (100%), craniofacial features, trunk and limbs, organ congenital malformations and neuropathy, cognition, and behavior. The dysmorphic appearance of neonatal individuals included thick eyebrows (100%), synophrys (40%), long eyelashes (100%), a long and shallow philtrum (100%), and downturned corners of a low‐set and malformed mouth. Abnormalities of the trunk and limbs included small hands (100%), hirsutism (90%), proximally placed thumbs, and small feet (50%). Congenital organ malformations include cryptorchidism (100%) in all boys, feeding difficulties (80%), gastroesophageal reflux (GERD) (70%), and hearing abnormalities (40%). A cleft palate was repaired in individuals 3 and 8, and a surgically corrected small jaw and repaired cleft palate improved dyspnea and feeding difficulties in individual 9. All individuals had intellectual disabilities. Individual 4 had an autism spectrum disorder (ASD) and displayed self‐injurious behavior and stereotypical movement, which led to difficulties with attending kindergarten. Individual 8 had symptomatic epileptic seizures but these were controlled with phenobarbital.

**Table 2:** Characteristic signs and symptoms and clinical auxiliary examination

		HPO ID	1	2	3	4	5	6	7	8	9	10
Growth	Prenatal growth^b^	0001511	+	+	+	+	+	+	+	+	+	+
	Postnatal natal growth retardation^b^	0004322	+	+	−	+	+	+	+	+	+	+
	Microcephaly^b^	0000252	+	+	+	+	+	+	+	+	+	+
Craniofacial features	Low anterior hairline	0000294	−	+	+	+	+	+	−	+	−	+
	Thick eyebrows^a^	0000574	+	+	+	+	+	+	+	+	+	+
	Synophrys^a^	0000664	+	+	−	−	+	−	−	+	−	−
	Long eyelashes	0000527	+	+	+	+	+	+	+	+	+	+
	Shortnose^a^	0003196	+	+	−	+	+	+	+	+	+	+
	Concave nasal ridge^a^	0011120	+	+	−	−	−	+	+	−	−	−
	Upturned nasal tip^a^	0000463	+	+	−	−	+	+	−	−	+	+
	Long, smooth philtrum^a^	0000343, 0000319	+	+	+	+	+	+	+	+	+	+
	Thin upper vermilion^a^	0000219	+	+	+	+	+	+	+	+	−	−
	Downturned corners of the mouth^a^	0002714	+	+	+	+	+	+	+	+	−	+
	Highly arched palate	0000218	+	−	−	+	−	+	+	−	−	+
	Cleft palate	0000175	−	+	+	−	+	−	−	+	+	−
	Abnormality of dentin	0010299	−	−	−	−	+	+	−	−	+	−
	Low‐set and malformed ears	0000369, 0000377	+	+	+	+	+	+	−	+	+	+
Trunk and limbs	Oligodactyly and adactyly (hands)^a^	0012165, 0009776	−	−	−	−	−	+	−	−	−	−
	Small hands^b^	0200055	+	+	+	+	+	+	+	+	+	+
	Proximally placed thumbs	0009623	+	+	−	−	+	−	−	+	+	−
	Clinodactyly or short fifth finger^b^	0004209, 0009237	+	+	+	+	+	+	+	+	+	+
	Abnormality of the palmar creases	0010490	−	+	+	+	+	+	−	−	+	−
	Small feet	0001773	−	+	−	+	+	−	+	+	−	−
	Abnormality of the phalanges of the toes	0010161	−	−	−	+	−	−	−	+	−	−
	Hirsutism^b^	0001007	+	+	−	+	+	+	+	+	+	+
	Congenital diaphragmatic hernia^a^	0000776	−	−	−	−	−	−	−	−	−	−
	Vertebral anomalies	0003468	−	−	−	−	−	−	−	−	+	−
Organ congenital malformations	Cryptorchidism	0000028	−	+	+	−	−	+	−	+	−	+
	Feeding difficulties	0011968	+	+	−	−	+	+	+	+	+	+
	Gastroesophageal reflux GERD	0002020	+	+	−	−	+	+	−	+	+	+
	Pectus excavatum	0000767	−	−	−	+	−	−	−	+	−	−
	Renal cyst	0001250	−	−	−	−	−	+	−	−	−	−
	Cardiovascular anomalies	0002564	−	−	−	−	−	−	−	−	+	−
	Ptosis	0000508	−	−	−	−	−	+	−	−	−	−
	Hearing abnormality	0000364	−	+	−	−	+	−	−	+	+	−
Neuropathy Cognition and behavior	Intellectual disability^b^	0001249	+	+	+	+	+	+	+	+	+	+
	Autism spectrum disorder (ASD)	0000729	−	−	−	+	−	−	−	−	−	−
	Self‐injurious behavior	0100716	−	−	−	+	−	−	−	−	−	−
	Stereotypic movements	0000733	−	−	−	+	−	+	−	−	−	−
	Seizure	0001250	−	−	−	−	−	−	−	−	+	−
Clinical auxiliary examination	Karyotype		46,XX	46,XY	46,XY	46,XX	46,XX	46,XY	46,XX	46,XY	46,XX	46,XY
	Cranial magnetic resonance		Normal	Normal	Normal	Normal	Normal	Normal	Normal	Normal	Normal	Normal
	Skeletal age		NA	NA	Normal	Normal	Normal	NA	Normal	NA	NA	NA
	Ultrasonic cardiogram		Normal	Normal	Normal	Normal	Normal	Normal	Normal	Normal	ASD*	Normal
	Free triiodothyroid acid (3.8–8.2 pmol/L)		NA	3.87	5.47	6.39	5.58	7.39	6.35	4.66	5.83	3.22
	Free thyroxine (12.1–22 pmol/L)		NA	12.19	13.86	15.27	14.25	15.94	13.54	17.71	14.09	8.53
	Thyroid stimulating hormone (0.85–6.5 uIU/ml)		NA	1.52	2.07	1.86	2.05	0.75	2.14	2.11	4.28	1.93
	Growth hormone		NA	NA	NA	2.04	1.56	1.37	1.84	NA	NA	NA
	Hemameba (3.5–9.5 × 10^9^/L）		11.09	13.09	5.59	11.01	9.56	10.93	10.88	7.29	8.73	5.96
	Erythrocyte (3.8–5.1 × 10^12^/L）		3.35	3.68	4.53	5.09	4.56	5.18	4.21	3.87	4.84	2.89
	Hemoglobin (120–140 g/L）		107	147	124	134	115	144	125	150	128	104
	Thrombocyte (125–350 × 10^9^/L）		262	234	171	171	125	241	215	35	320	211

Abbreviations: ASD*, Atrial Septal Defect; NA, not available.

^a^ Cardinal features for clinical features of Cornelia de Lange syndrome.

^b^ Suggestive features for clinical features of Cornelia de Lange Syndrome.

### 3.3 Clinical auxiliary examination

Table 2 lists the clinical supplementary examinations of the 10 individuals. The skeletal age was normal, and GH were subnormal in four individuals each. Echocardiography revealed an atrial septal defect (ASD) in individual 9. Individual 10 developed thrombopenia after undergoing anti‐infective therapy, and had thrombocyte counts between 35 and 111 × 10^9^/L, which were below normal. Levels of free triiodothyroid acid and free thyroxine were below normal in individual 10, but TSH was normal. The child was also transfused with erythrocytes on postnatal day 3 due to severe anemia.

### 3.4 Genetic analysis

We investigated variants in the individuals using whole exome targeted capture sequencing, and verified the results using Sanger sequencing and quantitative PCR (qPCR). Among 10 variants that we found in *NIPBL* (70%), *HDAC8* (20%), and *SMC3* (10%), six were novel (Table 3). The *NIPBL* gene contained two nonsense variants, c.7150C>T and c.2422C>T, three splicing variants, c.64+1G>A, c.6589+5G>A, and c.230+2T＞G, and two deletion variants, c.496delT and c.2492‐2493delAG. The *HDAC8* gene contained the missense variant, c.7150C>T, and an absence variant of 2.75 kb. The *SMC3* gene had the missense variant, c.1964G>T. According to the American Society of Medical Genetics and Genomics (ACMG) guidelines, 70% and 30% of these pathological variants were preliminarily determined as pathogenic and likely pathogenic, respectively. Individual 10 was diagnosed with CdLS based on her clinical characteristics. She carries a heterozygous deletion variant of 2.75 kb that included 8, 9 exon of the *HDAC8* gene.

**Table 3:** Genotype profile

Patient	Gene	RefSeq*	Exon	Nucleotide substitution	Amino acid substitution	Mutation type	dbSNP/1000G/EVS/ExAC	Inheritance	Origin of variation	Status	Pathogenicity
1	*NIPBL*	NG_006987.2, NM_133433.3	42	c.7150C＞T	p.Q2384X	nonsense	0/0/0/0	AD	*De novo*	PMID:20081834	Pathogenic
2	*NIPBL*	NG_006987.2, NM_133433.3	10	c.2422C＞T	p.R808X	nonsense	0/0/0/0	AD	*De novo*	PMID:20824775	pathogenic
3	*NIPBL*	NG_006987.2, NM_133433.3	–	c.64+1G＞A	–	splicing	0/0/0/0	AD	*De novo*	PMID:15591270	pathogenic
4	*NIPBL*	NG_006987.2, NM_015384	–	c.6589+5G＞A	–	splicing	0/0/0/0	AD	*De novo*	PMID:20824775	pathogenic
5	*NIPBL*	NG_006987.2, NM_015384	6	c.496delT	p.F166Lfs50	deletion	0/0/0/0	AD	*De novo*	Novel	pathogenic
6	*NIPBL*	NG_006987.2, NM_133433.3	10	c.2492‐2493delAG	p.E831Afs8	deletion	0/0/0/0	AD	*De novo*	Novel	pathogenic
7	*NIPBL*	NG_006987.2, NM_133433.3	3	c.230+2T＞G	–	splicing	0/0/0/0	AD	*De novo*	Novel	pathogenic
8	*HDAC8*	NG_015851.1, NM_018486.2	6	c.556C＞A	p.E186K	missense	0/0/0/0	XLD	*De novo*	Novel	Likely pathogenic
9	*HDAC8*	NG_015851.1, NM_018486.2	8–9	–	–	deletion	–	XLD	–	Novel	Likely pathogenic
10	*SMC3*	NG_012217.1, NM_005445	19	c.1964G＞T	p.G655V	missense	0/0/0/0	AD	*De novo*	Novel	Likely pathogenic

* The GenBank reference sequence shown in this Table are described using the version of GRCh37/hg19.

### 3.5 Medical follow‐up

Individuals 1 and 10 had feeding difficulties during the neonatal period and received milk via a nasogastric tube. However, the parents refused further treatment. The neonates choked on milk and died of asphyxia 1 month later.

Individual 2 was discharged from hospital after 1 month, but feeding difficulties persisted and his weight slowly increased. He was re‐admitted to hospital with aspiration pneumonia.

Individual 3 has been undergoing active rehabilitation intervention since birth. A cleft palate was repaired at the age of 1.5 years. At the age of 2 years, he underwent orchiopexy, then CdLS was diagnosed at the age of 3.5 years. At that time, his adaptability and fine motor skills were extremely delayed, while general movement, language, and behavior was moderate. After 1 year of rehabilitation, his scores for each category improved considerably, reaching moderate developmental retardation, and he was able to attend kindergarten with normal children. Surprisingly, this child reached height of 110 cm at the age of 5 years, which is the average value for normal children.

**Figure 1:**
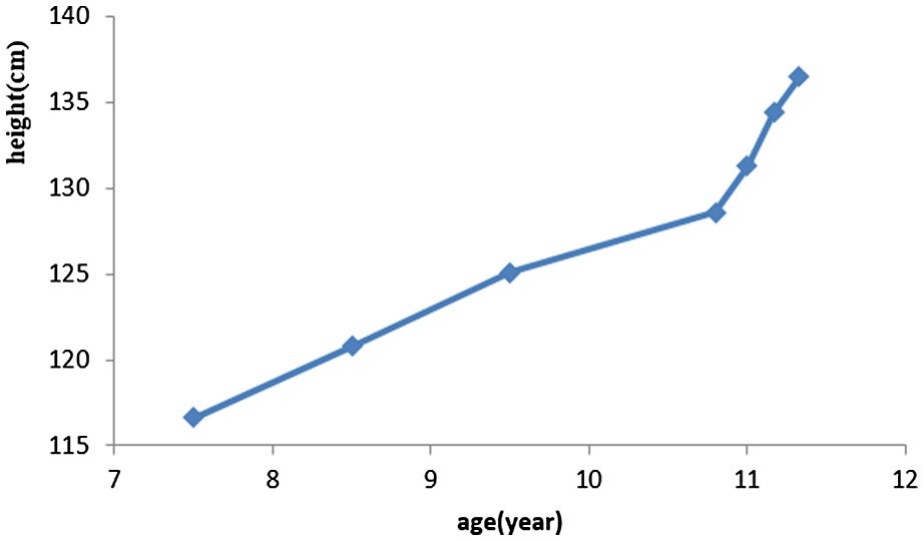
Height and age curve of individual 4 of CdLS

Individual 4 was born small for gestational age with persistently severe growth retardation (height −2.4 standard deviations [SD]) and mild dysmorphic features. She has attended the Pediatric Healthcare Institute of our hospital for treatment of developmental retardation since the age of 7.5 years. Her height at that time was 116.6 (−1.9 SD) cm, and her annual growth and development has been assessed since then. She reached 128.6 (−2.6 SD) cm by the age of 10.8 years, when genetic tests confirmed a diagnosis of CdLS. Her insulin‐like growth factor‐1(IGF‐1), cortisol, and adrenocorticotrophic hormone (ACTH) were normal, but levels of GH and GH stimulation test values were decreased. With the consent of her parents, she underwent therapy with nightly subcutaneous injections of growth hormone, 0.1 IU/kg. Her height was measured every 2 months, and we measured IGF‐1 as an evaluation index of rhGH effectiveness and safety. Her height increased by 5.2 cm to 136.5 cm within 6 months, and the SD increased by 0.6. Her IGF‐1 level was normal, and this individual remains on rhGH therapy, which continues to be effective. Figure 1 shows her growth curve.

Individual 5 was diagnosed with CdLS as a neonate, but she did not comply with follow‐up and treatment. She was repeatedly admitted to hospital with aspiration pneumonia due to a cleft palate and had extreme difficulties with feeding. At the age of 1 year, she weighed only 5 kg and was 68 cm long. Overall, growth and intellectual development were delayed, and she was unable to turn over, sit alone, or recognize her mother. She also had a serious hearing disorder and was fitted with a hearing‐aid to assist language training, but the effects were unsatisfactory. Low body weight and a small head circumference precluded installing a cochlear implant.

Individual 6 had extreme feeding difficulties, milk protein allergies, recurrent hematochezia, and severe eczema. Orchiopexy was completed at the age of 2 years. He was fed with whole amino acid milk until the age of 3 years. By 4 years of age, he could sit unaided, and 1 year later he could walk unaided, but was unable to speak. At 5 years of age, he was 85 cm tall, weighed 13.5 kg, and had serious growth retardation and intellectual disability. Nonetheless, his parents remain actively involved in his rehabilitation and training.

Individual 7 had extreme feeding difficulties. She was diagnosed with CdLS at the age of 1.5 years and was orally fed with high‐calorie (100 kcal/kg) milk. At the age of 3 years, she weighed 14 kg and was 81.5 cm (<2 SD), but she could walk unaided and express simple sentences, although her pronunciation of some words was unclear. Behavioral competence assessment showed a moderate developmental delay in adaptability, fine motor skills, general movement, language, and behavior. The family is in active recovery.

Individual 8 had serious issues with height and weight, remaining 75 cm (<SD) tall and weighing 7 kg until the age of 3 years. Brainstem auditory evoked potentials showed high‐frequency hearing impairment, so he was unable to speak. His intelligence and movement development were delayed. Although, the importance of surgery for the cleft palate and cryptorchidism was explained to his parents, they withheld consent to surgery.

Individual 9 underwent surgical small jaw correction and cleft palate repair, which improved her dyspnea and feeding difficulties. However, her weight and height remained at <2 SD. She was unable to walk unaided at the age of 2, which is a severe movement and intellectual disability. A severe hearing impairment rendered her unable to speak by the age of 3 years.

### 3.6 Comparison of individuals with CdLS from China and other diverse backgrounds

We summarized the clinical features of 41 Chinese individuals of CdLS, including two from the current study (Table 4). The cases were classified according to their gene mutation as *NIPBL* (83%), *HDAC8* (12%) or *SMC3* (2%), and SMC1A (2%). All individuals of Chinese CdLS displayed symptoms of growth deficiency (97%, 34/35); intellectual disability (100%, 21/21); and the typical craniofacial characteristics of CdLS, which include synophrys (65%, 24/37), long eyelashes (92%, 31/36), short nose (80%, 28/35), long, smooth philtrum (100%, 35/35), thin upper vermiliona (94%, 33/35), down‐turned corners of the mouth (87%, 26/30), and micromelia (100%, 31/31). All Chinese individuals diagnosed with CdLS also had major malformations, including cleft palates, crease abnormalities, hirsutism, gastroesophageal reflux, renal anomalies, cardiovascular anomalies, ptosis, hearing loss, and intellectual disability when comparing the phenotypes of Chinese individuals with CdLS with those of African, Asian, Latin American, and Middle Eastern descent, we identified significant differences in the clinical characteristics of the following symptoms: synophrys, long eyelashes, cleft palate, micromelia, crease abnormalities, gastroesophageal reflux, ptosis, and hearing loss (Table 1; *p* < 0.05; *χ* ^2^ test).

**Table 4:** Summary of the individuals with CdLS from China Compared with other diverse backgrounds

	Percentage in total of Chinese so far	Percentage of Africa in Dowsett and et al. (2019)	Percentage of Asia in Dowsett and et al. (2019)	Percentage of Latin America in Dowsett and et al. (2019)	Percentage of Middle East in Dowsett and et al. (2019)	*p*‐Value
Number	41	14	23	22	8	
*NIPBL*	83% (34/41)	100% (6/6)	75% (6/8)	77%	60% (3/5)	
*HDAC8*	12% (5/41)	0	25% (2/8)	18%	20% (1/5)	
*SMC3*	2% (1/41)	0	0	0	0	
*SMC1A*	2% (1/41)	0	0	5% (1/22)	20% (1/5)	
Growth deficiency	97% (34/35)	100% (13/13)	100% (18/18)	100%	80% (4/5)	0.066
Microcephaly	100% (36/36)	NA	NA	NA	NA	
Low anterior hairline	74% (23/31)	NA	NA	NA	NA	
Synophrys	65% (24/37)	100%	91%	100%	88%	0.001
Long eyelashes	92% (31/36)	100%	100%	100%	75%	0.023
Short nose	80% (28/35)	100%	83%	100%	88%	0.099
Long,smooth philtrum	100% (35/35)	100%	100%	100%	100%	0.999
Thin upper vermiliona	94% (33/35)	NA	NA	NA	NA	
Downturned corners of the mouth	87% (26/30)	NA	NA	NA	NA	
Cleft palate	38% (8/21)	23% (3/13)	11% (2/18)	27%	80% (4/5)	0.029
Micromelia	100% (31/31)	92% (12/13)	89% (16/18)	100%	60% (3/5)	0.004
Crease abnormalities	74% (17/23)	38% (5/13)	33% (6/18)	32%	20% (1/5)	0.01
Hirsutism	84% (21/25)	85% (11/13)	67% (12/18)	68%	100% (5/5)	0.322
Gastroesophageal reflux	70% (7/10)	85% (11/13)	28% (5/18)	73%	40% (2/5)	0.011
Renal anomalies	18% (2/11)	27% (4/13)	11% (2/18)	9%	20% (1/5)	0.506
Cardiovascular anomalies	38% (8/21)	28% (3/13)	22% (4/18)	41%	40% (2/5)	0.643
Ptosis	18% (2/11)	71%	39%	36%	0%	0.013
Hearing loss	60% (9/15)	69% (9/13)	22% (4/18)	32%	60% (3/5)	0.037
Intellectual disability	100% (21/21)	85% (11/13)	100% (18/18)	100%	80% (4/5)	0.053

## 4 DISCUSSION

### 4.1 Antenatal and neonatal features

Rare CdLS is characterized by the presence or absence of a remarkable variety of congenital deformities and complications (Mariani et al., 2016). A recent review of 53 individuals with CdLS indicated that about 25% of them are diagnosed prenatally (Clark et al., 2012). The minimum gestation period associated with classical CdLS is 15 +6 weeks, and the fetus harbored a novel de novo frameshift pathogenic variant of *NIPBL* with targeted sequencing of genes. The fetus described in that case report died *in utero*, and multiple congenital anomalies identified postmortem included IUGR, upper limb anomalies, a ventricular septal defect, a diaphragmatic hernia, and skeletal as well as genitourinary abnormalities. However, increased nuchal translucency and low maternal serum pregnancy‐associated plasma protein‐A were identified in a related prenatal screening (Hague, Twiss, Mead, & Park, 2019). The decreasing pregnancy‐associated plasma protein‐A value indicated CdLS, but further study was needed for confirmation. A review of CdLS between 1965 and 2007 showed that 7% of 426 individuals had been diagnosed with CdLS as neonates (Schrier et al., 2011). The present study found that 50% were diagnosed as neonates. Two of five with more severe growth restriction and intellectual disability than the others died 3 months after diagnosis. One woman developed an upper respiratory tract infection during the 3rd month of pregnancy, and the mother of case 2 had hypothyroidism. At the time of delivery, five of the women had polyhydramnios, which might have been associated with effects of a small jaw on swallowing function, and vestigation of a larger sample is needed.

### 4.2 Clinical malformations

The first international consensus on CdLS in 2018 proposed that the phenotype of CdLS comprises clinical signs and symptoms, and divided them into cardinal and suggestive features. The main cardinal features are (score of 2 for each if present): synophrys, short nose, concave nasal ridge, long and smooth philtrum, thin upper lip vermilion, hand oligodactyly, and a congenital diaphragmatic hernia. The suggestive features are (score of 1 for each if present): global developmental delay, prenatal/intrauterine growth retardation, microcephaly, small hands, short fifth finger, and hirsutism. The diagnosis of CdLS is determined based on the summed score (Kline et al., 2018). All 10 of our individuals scored ≥11. Cardinal dysmorphic features of these individuals included thick and long eyebrows (100%) and downturned corners of the mouth (90%), and suggestive features included microcephaly (100%), postnatal natal growth retardation (100%), intellectual disabilities (100%), small hands (100%), and hirsutism (90%). They also had long eyelashes (100%), and abnormal palmar creases (60%), which are included as new suggestive features. The main cardinal features of CdLS in our individuals were generally in accordance with those of other populations (Hei, Gao, & Wu, 2018; Kline et al., 2007). A recent review of CdLS manifestations in otolaryngology indicated that cleft and high‐arched palates affect 22.0% and 70.6% of such individuals (Bergeron, Chang, & Ishman, 2019). The present results are consistent with these. Loos, Wieczorek, Würtz, von der Malsburg, and Horsthemke (2003) analyzed 55 photographs of individuals with five genetic syndromes including 12 with CdLS, which was determined at a diagnostic accuracy of 76%. One year thereafter, a new facial dysmorphology analysis (FDNA) was developed (Basel‐Vanagaite et al., 2016; Loos et al., 2003) with 87% diagnostic accuracy for CdLS among 34 individuals. However, the nonspecific characteristics of CdLS can lead to a delayed diagnosis. In addition to craniofacial and limb dysplasia, individuals with CdLS have congenital malformations of multiple organs. A recent study uncovered a 67.5%–87% incidence of GERD (Macchini et al., 2010), and deformities of the digestive system in individuals with CdLS including pyloric stenosis, congenital diaphragmatic hernia, intestinal malrotation, volvulus, duodenal atresia, annular pancreas, anal atresia, Meckel diverticulum, and inguinal hernia (Kline et al., 2007). About 30% of individuals with CdLS have cardiac structural abnormalities, including pulmonary artery stenosis, ventricular, and atrial septal defects (Chatfield et al., 2012). Structural renal malformations in 40% of individuals with CdLS include pelvic dilation, renal dysplasia and bladder and ureteral reflux, which could affect the renal function (Boyle et al., 2014). Malformations of the reproductive system include cryptorchidism in 82%, micropenis in 37%, and hypospadias 9% of boys with CdLS. About 20% of individuals with CdLS develop epilepsy, and partial epilepsy was the most prevalent (Verrotti et al., 2013). Conductive and sense otoneurogenic hearing are often impaired (Marchisio et al., 2014). Our 10 individuals with CdLS had feeding difficulties (80%), GERD (70%), cardiovascular anomalies (10%), renal cysts (10%), cryptorchidism (100%) in boys, ptosis (10%), and seizures (10%). Chinese individuals with CdLS displayed symptoms that included growth deficiency; short nose; long, smooth philtrum; hirsutism; renal anomalies; cardiovascular anomalies; and intellectual disability. When compared with the phenotypic characteristics of individuals with CdLS reported by Dowsett et al. (2019), there were significant differences in the clinical characteristics of the following symptoms: synophrys, long eyelashes, cleft palate, micromelia, crease abnormalities, gastroesophageal reflux, ptosis, and hearing loss. However, the sample size used for comparison was small and many of the clinical descriptions lacked detail. Thus, a larger sample is needed for further investigation.

### 4.3 Growth retardation and follow‐up

After a diagnosis of CdLS has been clinically confirmed, multidisciplinary collaboration is required for further evaluation and treatment because CdLS involve multiple system abnormalities. Common internal organ abnormalities should be screened, then corrected in timely fashion. Thereafter, growth and development should be assessed. Standard growth curves of CdLS prepared based on data from 180 individuals with clinically confirmed CdLS in 1993 confirmed that weight, head circumference, and height were all below the 5th percentile at birth, and during infancy, adolescence, and adulthood (Kline, Barr, & Jackson, 1993). If growth is restricted, then nutrition, gastrointestinal dysfunction, and related hormone levels (thyroid dysfunction and GH) should be assessed (Ansari et al., 2014). One study found that 17 of 27 individuals with CdLS had IUGR, and 4 of 12 had abnormal endocrine hormones (Kousseff, Thomson‐Meares, Newkirk, & Root, 1993). One case report describes that GH therapy obviously improved the height of an individual with CdLS (de Graaf et al., 2017). Skeletal age was normal in 4 of 10 individuals examined in the present study, but GH levels were subnormal for four others. Individual 4 at the age of 10.8 years was 128.6 (SD −2.6) cm tall, so she underwent GH therapy. Her height increased by 5.2 cm to 136.5 cm with 6 months, and the SD increased by 0.6. She is only the second known child with CdLS to benefit from this therapy to date. In addition, GERD should be assessed with pH monitoring and endoscopy, and if diagnosed, active treatment and follow‐up are required.

Most individuals with CdLS have severe to moderate intellectual disabilities and invariably, delayed motor development (Kline et al., 2018; Mariani et al., 2016). A study of 51 individuals with CdLS showed that at 5 years of age, 99%, 63%, and 38% of them could sit unaided, walk unaided, and speak (38%) (Huisman et al., 2017). Seizures are common in CdLS, in particular, when accompanied by *SMC1A* variants (Pavlidis, Cantalupo, Bianchi, Piccolo, & Pisani, 2014). Among 168 publications describing ASD phenomenology, 43% and 56% of individuals with CdLS have ASD, and exhibit self‐ injurious behavior, respectively, especially, of the hand‐directed type (Oliver, Sloneem, Hall, & Arron, 2009; Richards, Jones, Groves, Moss, & Oliver, 2015). Only one of our individuals was autistic, and self‐injurious, mainly involving upper limb movement. No specific magnetic resonance imaging (MRI) changes were found in 15 individuals with CdLS (Roshan Lal et al., 2016), with which the present study was consistent. The microcephaly phenotype in CdLS confirmed no indication for molecular neuroimaging (van den Berg et al., 2017). Standardized follow‐up, effective surgery, and standardized rehabilitation and nursing can improve the quality of life of children with CdLS. Communication skills should be optimized, and speech therapy is highly recommended within the first 18 months of life (Kline et al., 2018; Romski et al., 2010). The study found that cleft palate repair and small jaw correction greatly diminished the feeding difficulties and promoted the growth. Frequent respiratory infections are thought to be associated with laryngeal deformities, hypotonia and uncoordinated swallowing, GERD, and immunological anomalies (Jyonouchi, Orange, Sullivan, Krantz, & Deardorff, 2013).

If most individuals with CdLS receive appropriate care especially during the first 2 years after birth, they can survive into adulthood. However, their lifespan will be 10–20 years shorter than that of the general population, depending on timely and appropriate medical intervention and the severity of malformations. One study of 295 individuals with CdLS found that the most common causes of perinatal death were congenital diaphragmatic hernia (>33%), followed by congenital heart disease (17%) and respiratory diseases (13%). The most common causes of infant death were respiratory problems (35%), cardiovascular diseases (27%), and gastrointestinal problems (18%), followed by sepsis (4%) and central nervous system problems (4%). The main causes of death in preadolescent children and adolescents were respiratory problems (32%), gastrointestinal problems (18.8%), accidents and cardiovascular diseases (10.2%), and central nervous system diseases (9%) (Ansari et al., 2014; Coppus, 2013; Schrier et al., 2011). Two (20%) of our individuals died within 3 months, because of difficulties in feeding and respiratory failure, which was consistent with other studies. However, due to insufficient understanding and a disregard for CdLS, individual history can be unreliable and follow‐up examinations can be missed. Thus, treatment delays and complications are more prevalent among individuals with, than without CdLS. A First Aid booklet containing individual of CdLS information and personalized programs has been recommended (Ailey, Brown, & Ridge, 2017).

### 4.4 Cohesin complex

**Figure 2:**
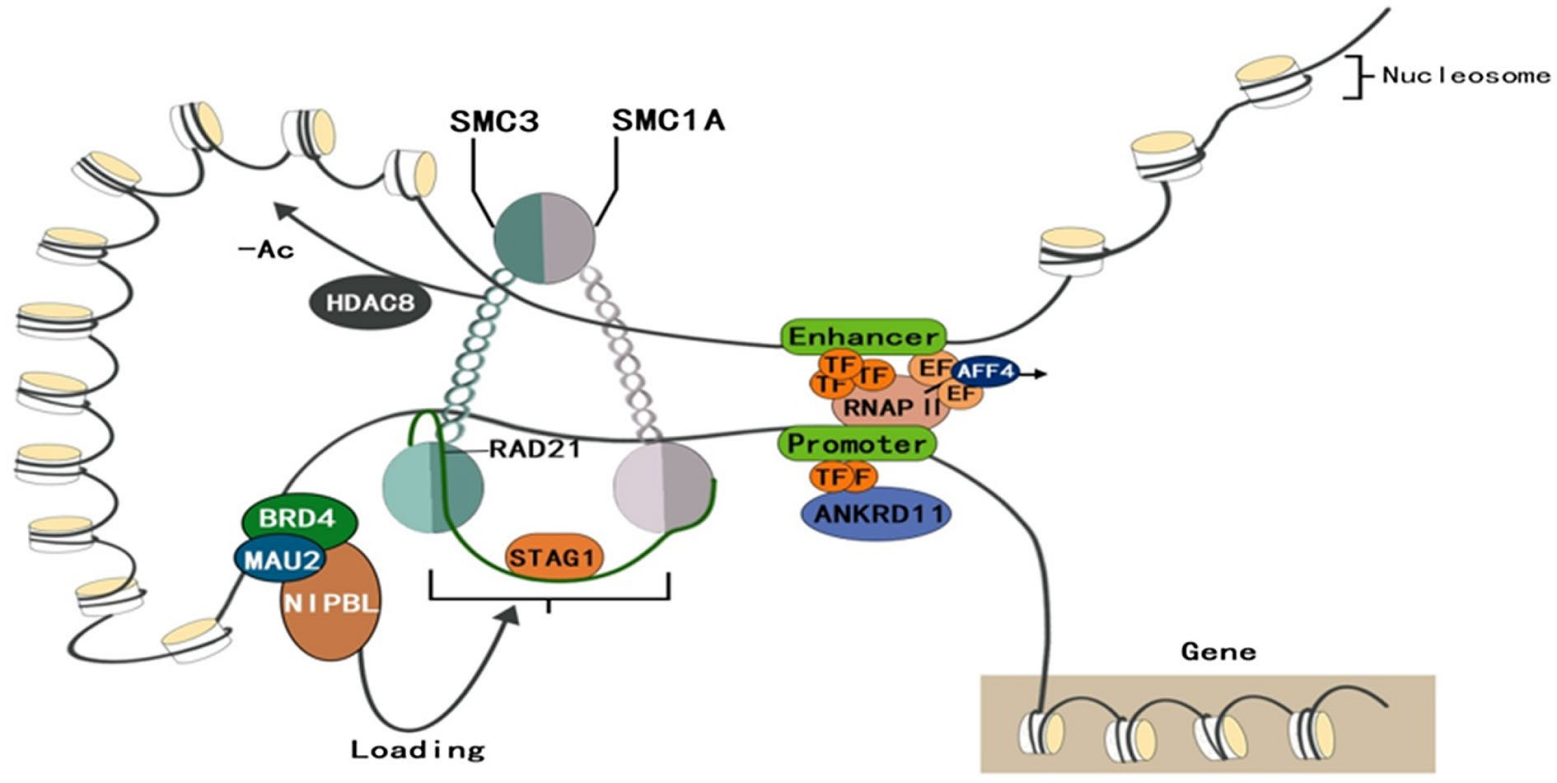
Cohesin complex. Cornelia de Lange syndrome (CdLS) is a cohesinopathy. CdLS is caused by genetic variants that affect the subunits or regulators of the cohesin complex. The structural core components *RAD21*, *SMC1A*, and *SMC3* of cohesin are thought to form a tripartite ring entrapping chromatids. In humans, cohesin subunit SA1 (STAG1), STAG2, or STAG3 directly attach to the ring and form part of the core complex *NIPBL* and MAU2 chromatid cohesion factor homolog form a heterodimeric complex named kollerin that is required for cohesin loading onto DNA, and in which BRD4 interacts with *NIPBL*. *HDAC8* regulates the cohesin complex release from chromatin by deacetylating *SMC3*. Ac, acetyl group; AFF4, AF4/FMR2 family member 4; EF, elongation factor; RNAPII, RNA polymerase II; TF, transcription factor

Pathological variants of the cohesin complex have been associated with the CdLS spectrum at the molecular level, which subsequently alters the regulation of gene expression during development (Kline et al., 2018) (Figure 2). Cohesive proteins are important regulatory factors that mainly separate chromosomes, maintain genomic stability and chromatin structures, and regulate gene expression (Kamada & Barillà, 2018). However, the exact pathological mechanism of CdLS is not fully understood. Screening individuals with classical, nonclassical, and overlapping phenotypes led to the discovery of the CdLS pathogenic genes, *NIPBL*, *SMC1A*, *SMC3*, *RAD21*, *BRD4*, *HDAC8*, and *ANKRD11* (Huisman, Redeker, Maas, Mannens, & Hennekam, 2013; Kline et al., 2018).

The main function of the protein encoded by the cohesin complex gene is pathogenicity. The structural core components *RAD21*,* SMC1A*, and *SMC3* of cohesin are thought to form a tripartite ring entrapping chromatids. *NIPBL* is required for loading cohesin onto chromosomes (Muto et al., 2014). The chromatin‐related protein *BRD4*, enhances *NIPBL* loading by binding to acetylated histone H3 Lys27 and targeting the enhancer cluster (Hnisz et al., 2013). *HDAC8* regulates the release of cohesin complex from chromatin by deacetylating *SMC3* (Kline et al., 2018).

### 4.5 Genotypes and phenotypes

An analysis of mutational data from 278 individuals in several countries during 2003 revealed a close relationship between the genotype and clinical phenotype of CdLS. Nonsense, splice site, and truncation variants of *NIPBL* can result in more severe clinical phenotypes, but splice site and missense variants, as well as small frame deletions of *NIPBL*, are associated with a mild phenotype. Since the protein region is also an important factor affecting the clinical presentation of CdLS, even missense variants in the HEAT domain of *NIPBL* result in severe clinical phenotypes. (Mannini et al., 2013). The clinical phenotypes are more atypical among CdLS probands harboring *SMC3*/*SMC1A*, than *NIPBL* variants. However, to date, *SMC3* variants encoding another component of the cohesin complex, are typically missense changes, suggesting that loss‐of‐function variants are not tolerated (Gil‐Rodríguez et al., 2015; Olley et al., 2018). Individual 10 in the present study had mild craniofacial features and limb deformities that were accompanied by congenital heart disease and thrombocytopenia. Variations in the *HDAC8* phenotype are remarkably nonclassical and wide, but distinctive features of affected individuals in addition to those of CdLS include a large anterior fontanel, orbital hypertelorism, and happy personalities (Kaiser et al., 2014). Most female individuals with pathogenic *HDAC8* heterozygosity have a significant tendency toward X inactivation of the wild‐type allele (Kaiser et al., 2014; Parenti et al., 2016). Individual 8 in the present study was a boy with a missense variant, but he had a cleft palate and a large anterior fontanel. By the age of 3 years, the fontanel had still not closed, and his overall development, including height, movement, language, and intelligence, was delayed. Exons 1–9, 1–4, 5–10, and 11 are absent from the *HDAC8* gene in individuals with CdLS (Kaiser et al., 2014). Individual 9 was a girl with a typical phenotype due to the absence of two exons in *HDCA8*. Despite the active completion of surgery and rehabilitation, but she remains lagging overall.

### 4.6 *NIPBL* review

The *NIPBL* gene is located in 5p13.2, which is homologous to fly Nipped‐B and yeast SCC2 (sister chromatid cohesion 2), and consists of 47 exons that encode Nipbl protein (delangin) isoforms A and B with 2804 and 2697 amino acids, respectively (Krantz et al., 2004; Tonkin, Wang, Lisgo, Bamshad, & Strachan, 2004). The HGMD® accessed during October 2019 revealed a total of 445 *NIPBL* mutations, including missense/nonsense 174 (39%), small deletions 97 (22%), splicing 73 (16%), small insertions 42 (9%), gross insertions/duplications 9 (2%), small indels 7 (1.5%), complex rearrangements 2 (0.4%), and regulatory 1 (0.2%) types.

**Figure 3:**
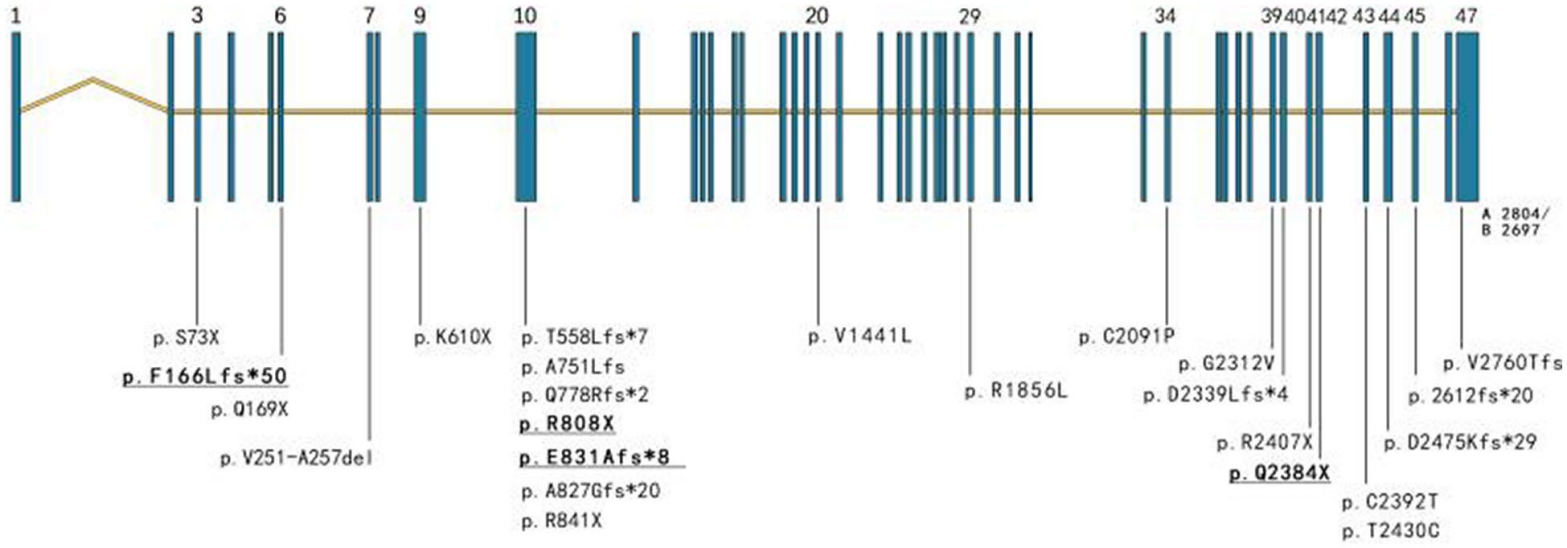
The pathological variants in *NIPBL* in Chinese. The underline is the new variants in the report

The protein Nipbl encoded by *NIPBL* is widely expressed in fetal and adult tissues, and in the zebrafish model, it obviously accumulates in regions involved in patterning the skeleton and soft tissues of the limbs, jaw, and face (Krantz et al., 2004). *NIPBL* contains an N‐terminal MAU interaction domain, a glutamine‐rich domain, a predicted nuclear‐localization signal (NLS), an undecapeptide repeat, and an importantly, a functional conserved domain with five HEAT repeats that are responsible for interactions with other proteins, participation in the chromatid aggregation process and the long‐term role of promoter enhancers (Yan et al., 2006). The SCC2 (Nipbl)/SCC4 loaded complex promotes the specific binding of the cohesin complex to chromosomes. The cohesin complex is loaded onto the Nipbl binding site, then slid along the chromosomal arm to more stable sites as transcription extends (Bermudez et al., 2012). A reduction in gene expression of ~15% results in the CdLS phenotype (Newkirk et al., 2017). In addition to data acquired from six individuals in the present study, 33 pathological variants identified in China to date (last access: October 2019) include missense 6 (18%), nonsense 7 (24%), small deletion 8 (24%), splicing 8 (24%), and small insertions/duplications 4 (12%) according to PubMed, ACMG guidelines, Wanfang Data, and the China National Knowledge Infrastructure (Figure 3). The phenotypes did not differ according to ethnicity or nation.

## 5 CONCLUSIONS

The clinical data and pathological variants of CdLS in Chinese individuals were like those in other populations. Reasonable rehabilitation, GH intervention, and surgical treatment of deformities can improve the prognosis of individuals with CdLS. All infants or children diagnosed with CdLS will need lifelong medical support, multidisciplinary therapy, and social care. Considering the few reports of Chinese individuals with CdLS and the absence of standardized treatment and follow‐up, further investigation of Chinese individuals with CdLS is needed to determine genotype‐phenotype correlations, follow‐up strategies, and appropriate care according to country characteristics.

## CONFLICT OF INTEREST

All authors in this study declare that they have no conflict of interest.
